# Feasibility, safety and outcomes of a virtual ward with remote monitoring for patients awaiting urgent coronary artery bypass graft surgery

**DOI:** 10.1136/openhrt-2025-003568

**Published:** 2026-02-20

**Authors:** Arun Kirupananthavel, Simon Woldman, Daniel A Jones, Gordon Ferguson, Charles Knight, Mick Ozkor, Stephen Edmondson, Andrew Archbold, Jason Radley, Cora Niblock, Anthony Johnson, Jade Theisinger, Alessia Rossi, Emad AlJaaly, Mihir Kelshiker, Nicholas S Peters, Kulvinder Lall, Debashish Das, Martin T Yates

**Affiliations:** 1 Barts Health NHS Trust, London, UK; 2 Barts Heart Centre, St Bartholomew’s Hospital, London, UK; 3 Centre for Cardiovascular Medicines and Devices, Queen Mary University of London, London, UK; 4 Barts Heart Centre, Barts Health NHS Trust, London, UK; 5 Barts and The London NHS Trust, London, UK; 6 St Bartholomew’s Hospital, London, UK; 7 General and Invasive Cardiology, Barts Heart Centre, St Bartholomew’s Hospital, London, UK; 8 Mid and South Essex NHS Foundation Trust, Basildon, UK; 9 Imperial College Healthcare NHS Trust, London, UK; 10 Department of Cardiac Engineering, Imperial College London, London, UK; 11 St Mary's Hospital and Imperial College London, London, UK; 12 Cardiology, Barts Health NHS Trust, London, UK

**Keywords:** Coronary Artery Bypass, Acute Coronary Syndrome, Telemedicine

## Abstract

**Background:**

Delays in performing urgent coronary artery bypass graft (CABG) surgery are increasing across the UK, with national wait times now exceeding guideline targets. Prolonged preoperative admissions contribute to hospital bed pressures, increased costs and negative psychosocial effects for patients. Virtual wards using remote patient monitoring (RPM) may enable safe early discharge for clinically stable patients awaiting surgery.

**Objectives:**

To evaluate the feasibility, safety and outcomes of a virtual ward pathway using RPM for patients awaiting urgent CABG surgery.

**Methods:**

A prospective, multicentre, single-arm study was conducted across three UK cardiac centres (December 2022–May 2025). Eligible patients were discharged home with daily symptom reporting via a digital platform and structured clinician review. The primary outcome was preoperative major adverse cardiovascular events (MACE). Secondary outcomes included 30-day mortality, resternotomy, time to surgery, postoperative stay, readmissions and patient experience.

**Results:**

128 patients were enrolled (mean age 61 years; 87% male). No preoperative MACE occurred (0%; 95% CI 0.0% to 2.3%). 30-day mortality was 0% (95% CI 0.0% to 2.9%), and resternotomy occurred in 2.3%, comparable to national rates. Median time from discharge to surgery was 10 days, saving an estimated 1152 inpatient bed-days. Postoperative length of stay was 7.0 days compared with a national average of 8.0 (p=0.084). Patient experience was favourable: 95% felt safe at home, and 89% found the platform easy to use.

**Conclusions:**

A virtual ward pathway with remote monitoring for selected patients awaiting urgent CABG was safe, feasible and associated with high patient acceptability and major reductions in inpatient utilisation. These findings support this model as a scalable approach to managing urgent surgical pathways while preserving safety and surgical timelines.

**WHAT IS ALREADY KNOWN ON THIS TOPIC**

- Delays in urgent coronary artery bypass graft (CABG) are increasing across the UK, with many centres unable to meet guideline-recommended timelines, leading to prolonged inpatient stays, increased costs and patient distress. While remote monitoring is established as safe and effective in other cardiac conditions, its role in the urgent CABG pathway has not previously been evaluated.

**WHAT THIS STUDY ADDS**

- This multicentre study demonstrates that a virtual ward with remote monitoring for selected urgent CABG patients is feasible and safe, with no preoperative major cardiovascular events and perioperative outcomes comparable to national audit data, while substantially reducing inpatient bed use and achieving high patient satisfaction.

**HOW THIS STUDY MIGHT AFFECT RESEARCH, PRACTICE OR POLICY**

- These findings support virtual wards as a scalable, real-world solution for managing selected urgent CABG patients in the community, aligning with National Health Service elective recovery priorities and informing future service design and commissioning decisions.

## Introduction

 The inpatient wait time for urgent coronary artery bypass graft (CABG) surgery has been increasing across the UK, posing significant challenges for both patients and the healthcare system. Urgent CABG is typically indicated for patients with acute coronary syndromes, including non-ST elevation myocardial infarction (NSTEMI) and unstable angina, where timely surgical intervention is critical for preventing further cardiac damage and improving survival outcomes. However, the 2025 National Adult Cardiac Surgery Audit highlights a decline in the timeliness of urgent CABG procedures, with no hospital meeting the target of performing CABG within 7 days of coronary angiography.^1^ The national average wait time for urgent CABG in England is now 14 days, with delays extending to 27 days in some hospitals.^1^

Extended wait times for CABG surgery increase risks to patient health and impose burdens on hospital resources. Prolonged hospitalisation raises the likelihood of hospital-acquired infections and complications related to immobility.^2^ Furthermore, prolonged separation from family and familiar environments can have psychosocial impacts, contributing to anxiety, depression and reduced well-being among patients awaiting surgery.^3^ Financially, longer hospital stays increase bed occupancy, contribute to inefficiencies such as bed blocking and increase costs for the National Health Service (NHS), adding strain to an already stretched healthcare system.^4^

For patients with NSTEMI who require revascularisation with CABG, clinical guidelines recommend that timing should be determined on an individualised basis, balancing patient stability, anatomical complexity and institutional resources.^5^ While earlier European Society of Cardiology guidance suggested that surgery be performed during the same hospital admission for select high-risk anatomical subsets, such as left main or three-vessel disease involving the proximal left anterior descending artery, this is not a universal recommendation.^6^ In the absence of robust randomised data, optimal timing remains case dependent. However, the growing surgical backlog has made timely intervention increasingly difficult, underscoring the need for innovative pathways that preserve patient safety while improving system efficiency.

Virtual wards, enabled by remote patient monitoring (RPM), represent a potential strategy to mitigate these challenges. By leveraging digital symptom reporting and clinician oversight, this approach may allow stable, low-risk CABG candidates to await surgery at home while maintaining surveillance for clinical deterioration. In cardiology, RPM has demonstrated efficacy in heart failure and post-percutaneous intervention (PCI) care,^7 8^ yet its application in the preoperative CABG setting remains underexplored.

This study evaluates the feasibility, safety and clinical outcomes of a virtual ward pathway for patients awaiting urgent CABG. We hypothesise that early discharge with RPM will:

1. Reduce inpatient stay duration without compromising safety.
2. Shorten postoperative hospital stay potentially via reduced preoperative deconditioning and preserved functional reserve.
3. Improve healthcare efficiency by reclassifying urgent cases as early electives.

This alternative care pathway may offer a scalable solution to the escalating pressures on cardiac surgical services.

## Methods

### Study design and setting

We conducted a regional, prospective cohort study across three centres: Barts Heart Centre, the highest volume cardiac surgical unit in the UK; Basildon Hospital; and Hammersmith Hospital.^1^ The study evaluated the feasibility and safety of a virtual ward pathway for patients awaiting urgent CABG using RPM to enable early discharge.

### Patient selection

Consecutive patients referred for urgent inpatient CABG (defined as requiring surgery during the index admission but not requiring immediate intervention) were screened for eligibility. Eligibility criteria (see online supplemental tables 1 and 2) included cardiovascular stability, absence of high-risk features (eg, severe left ventricular dysfunction or refractory symptoms) and ability to engage with the digital platform. As this was a pragmatic quality improvement (QI) service, onboarding to the virtual ward was offered at the treating consultant’s discretion and with patient agreement; accordingly, not all eligible patients were onboarded.

### Virtual ward pathway

Eligible patients were enrolled onto the Ortus-iHealth RPM platform, a secure, NHS-compliant digital portal enabling real-time symptom reporting and clinician communication. The pathway comprised:

1. Predischarge consultation: clinicians provided structured education on RPM use, emphasising daily symptom logging.
2. Early discharge: patients were discharged with a scheduled CABG surgery date within 10 days and instructed to submit daily symptom reports via the portal.
3. Readmission: patients returned 24 hours preoperatively for standard assessments.

Patients were admitted to the virtual ward and submitted a once daily fixed response symptom check-in via a secure smartphone app or web portal, covering chest pain, palpitations and presyncope (the full questionnaire is provided in online supplemental appendix 1). The questionnaire was deliberately calibrated for high sensitivity to capture early signs of deterioration.

A specialist cardiac surgery nurse reviewed the virtual ward and submissions once daily and, using prespecified red flag criteria, initiated same-day telephone triage and escalation to the on-call cardiac surgery consultant when indicated. Non-submission prompted proactive same-day phone follow-up to confirm well-being and assist completion. Escalation actions included safety netting advice, arranging urgent clinic or hospital review or bringing surgery forward through established pathways. Both smartphone and computer access were supported; usability issues identified during follow-up were logged and addressed contemporaneously.

### Outcomes

Data were collected on both primary and secondary endpoints.

Primary endpoint:

1. Preoperative major adverse cardiovascular events (MACE) from discharge to virtual ward to index CABG or censoring, defined as myocardial infarction, stroke or cardiovascular death.

Secondary endpoints:

1. All-cause mortality and resternotomy within 30 days after CABG, compared with national benchmarks.
2. Time from discharge to surgery and time from angiography to surgery.
3. Postoperative length of stay.
4. Preoperative safety events (non-MACE): monitoring-triggered escalations and unplanned readmissions.
5. Inpatient bed-days saved and service efficiency, including conversion of urgent inpatients to early electives.
6. Patient satisfaction.

### Statistical analysis

Preoperative MACE was analysed as a binomial proportion over the monitored preoperative period (virtual ward discharge to index CABG or censoring). Exact (Clopper-Pearson) 95% CIs were reported; for zero events, only the upper 95% bound is shown. No hypothesis testing was performed given the single-arm design.

To evaluate the comparability of study outcomes with national benchmarks, we applied statistical methods appropriate to each endpoint. For resternotomy rates, equivalence testing was performed using the two one-sided tests procedure with a predefined equivalence margin of ±0.5% around the national average of 2.2%.^1^ Z-scores were calculated comparing the observed rate to the margin boundaries, and one-sided p values >0.05 indicated statistical equivalence within this margin.

For 30-day all-cause mortality, exact binomial CIs and one-sided exact binomial tests were used to assess whether the observed mortality rate was consistent with the national benchmark of 0.99%.^1^

For postoperative length of stay, a one-sample t-test compared the study cohort’s mean length of stay against the national average of 8 days.^1^ In the absence of a reported SD for the national data, a clinically reasonable estimate of 1.5 days was assumed. A two-sided p value <0.05 was considered indicative of a statistically significant difference.

### Ethics

This study was conducted as a QI project, aimed at enhancing patient care and operational efficiency within the cardiac surgery department. As a QI project, formal ethical approval was not required; however, the project team adhered to rigorous ethical standards in line with institutional policies. Patient consent was obtained for participation, with assurances regarding data confidentiality and secure storage on the Ortus-iHealth platform. Patients were informed that they could withdraw from the remote monitoring pathway at any time without impacting their clinical care.

### Patient and public involvement

Patients were not formally involved in the design, conduct or dissemination planning of this QI study. However, patient feedback was actively collected via satisfaction surveys during the intervention period to inform acceptability and safety perceptions of the virtual ward pathway. Future iterations of this pathway will include structured patient and public involvement to guide refinement of outcome measures and dissemination strategies.

## Results

### Study population

Between December 2022 and May 2025, a total of 128 patients were enrolled in the remote monitoring virtual ward pathway following referral for urgent CABG. The cohort had a mean age of 61 years, and 87% were male (table 1). Most patients presented with acute coronary syndromes: NSTEMI (55%), ST elevation myocardial infarction (15%), unstable angina (12%) and stable angina (18%); troponin on admission was positive in 78% of patients (table 2). The prevalence of comorbidities was high: hypertension (63%), hypercholesterolaemia (61%), diabetes (41%) and a history of smoking (45%) (table 1).

**Table 1:** Demographics

Age, years	61.0±9.0
Sex	
Male	111 (87%)
Female	17 (13%)
Clinical history	
Diabetes	52 (41%)
Smoking history	58 (45%)
Hypertension	80 (63%)
Hypercholesterolaemia	78 (61%)

**Table 2:** Clinical presentation

Angina	23 (18%)
Unstable angina	15 (12%)
NSTEMI	68 (55%)
STEMI	18 (15%)
Troponin positive	90 (78%)

NSTEMI, non-ST elevation myocardial infarction; STEMI, ST elevation myocardial infarction.

Primary and secondary outcome measures are summarised in table 3.

**Table 3:** Key outcome metrics

Median time from discharge to surgery (days)	10 (IQR 7–13)
Median time from angiography to surgery (days)	13 (IQR 10–17)
Median preoperative stay (days)	1 IQR (1–1)
Median postoperative hospital stay (days)	5 IQR (5–7)
Preoperative MACE	0, 0%
30-day mortality	0, 0%
Resternotomy for bleeding	3, 2.3%
Unplanned readmissions	5, 3.9%

MACE, major adverse cardiovascular events.

### Primary endpoint

Preoperative MACE (myocardial infarction, stroke or cardiovascular death) from discharge to virtual ward to index CABG.

Zero events occurred during monitoring (0/128); the exact binomial 95% CI was 0.0% to 2.3%. This suggests no observed harm in the preoperative period, acknowledging limited precision for rare events.

### Secondary endpoints

#### 30-day mortality and resternotomy

Zero deaths occurred within 30 days post-CABG (0/128). The exact binomial 95% CI for mortality ranged from 0.0% to 2.9%, consistent with the national benchmark mortality rate of 0.99% (p=0.28, one-sided exact binomial test).

Resternotomy occurred in 3/128 (2.3%, 95% CI 0.49% to 6.70%). This did not differ from the national rate of 2.2% (two-sided exact test, p=0.76). Formal equivalence within narrow margins (±0.5%) was not supported by the sample size.

#### Time to surgery

The virtual ward pathway enabled timely surgical access following discharge. The median time from hospital discharge to surgery was 10 days (IQR: 7–13). The median time from coronary angiography to surgery was 13 days (IQR: 10–17).

#### Postoperative length of stay

The mean postoperative length of stay was 7.0 days (SD 6.5). A one-sample t-test comparing this to the national average of 8.0 days yielded a t-statistic of –1.74 with 127 df (p=0.084). This difference was not statistically significant at the conventional threshold of p<0.05.

#### Preoperative escalation and unplanned readmissions

Throughout the monitoring period, 92.8% of patients completed at least one symptom questionnaire, yielding 955 submissions, indicating high engagement, though some daily entries were missed and a minority of patients did not engage. The patients who did not engage with the digital platform were monitored with daily phone calls.

**Figure 1:**
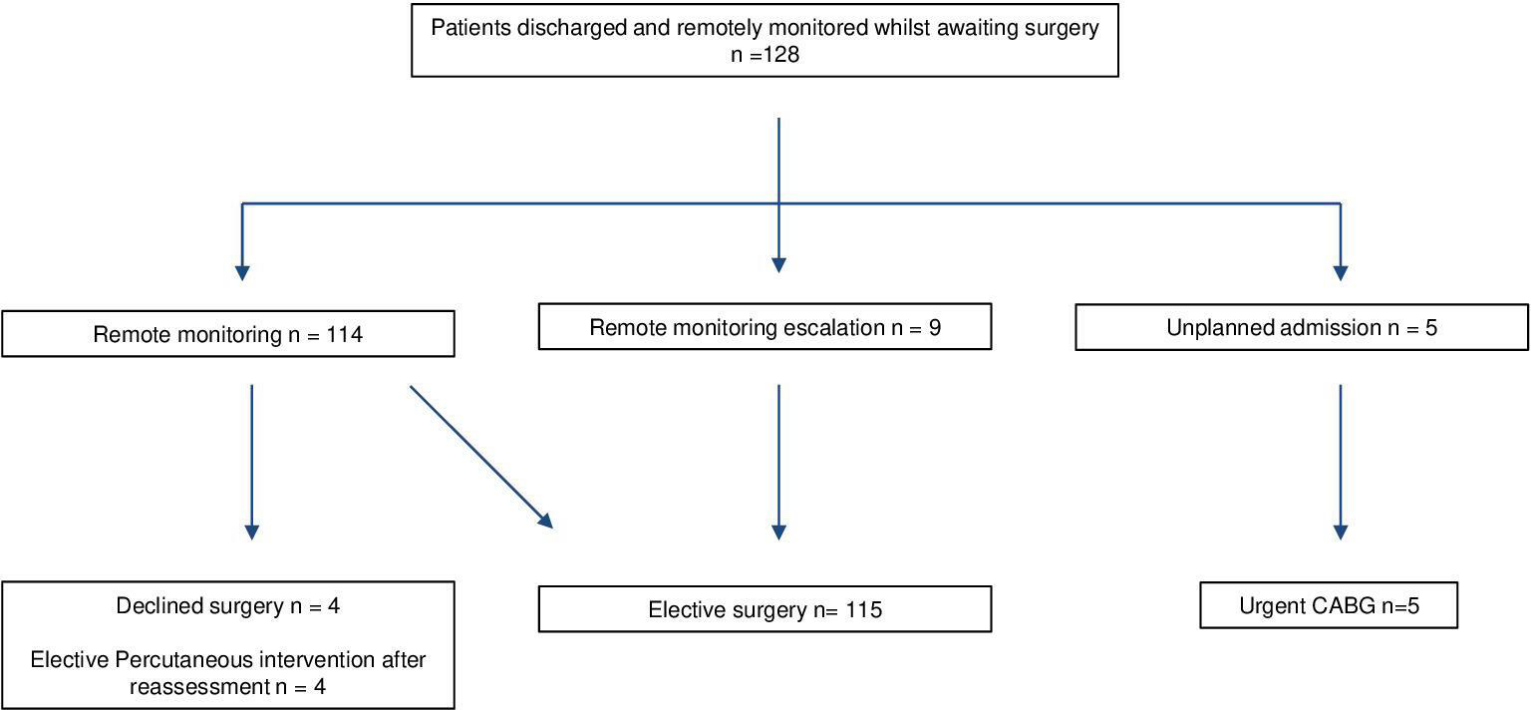
Overview of patient flow through the virtual ward pathway from referral to surgery. CABG, coronary artery bypass graft.

The platform generated 65 red alerts from 34 unique patients; in nine cases (7% of total patients), these alerts prompted escalation of surgical scheduling. After onboarding, four patients opted out of surgery, and four underwent elective PCI following reassessment. There were five unplanned readmissions and all five proceeded to urgent CABG. An overview of the patient pathway is illustrated in figure 1.

Not all red alerts led to escalation of surgical priority. The high-sensitivity questionnaire was intended to trigger same-day clinician review by telephone; after this assessment, often with discussion with the on-call cardiac surgery consultant, a decision was made on safety netting, urgent review if needed, or proceeding with the planned elective pathway.

Of the five unplanned readmissions (3.9% of total patients), four were cardiac presentations and one non-cardiac. Three of the patients triggered red alerts on the system due to chest pain but self-presented to the hospital before the clinician could make contact. One patient developed chest pain between scheduled symptom entries and self-presented to the hospital. The remaining patient had a non-cardiac presentation (headache) but stayed for CABG during the same admission. None of these readmissions led to delays in care or evidence of harm. An overview of the pathway is shown in figure 1.

#### Inpatient bed-days saved and system efficiency

The median preoperative hospital stay was 1 day. The median time from discharge to surgery was 10 days. Based on this, the estimated number of inpatient bed-days saved was 1152 days across the cohort (128 patients×9 days saved per patient).

Each patient was onboarded by a clinician, who spent an average of 10 min explaining the pathway, the digital monitoring system and the steps leading to surgery. Daily reviews through the digital platform took an average of 30 seconds per patient. In the case of red flags (signifying potential patient deterioration), the clinician spent an average of 10 minutes managing the patient’s care.

#### Patient satisfaction

Patient-reported outcomes were strongly favourable. 95% of patients reported feeling safe at home while awaiting surgery, and 96% stated that early discharge did not result in surgical delays. The remote monitoring platform was well received, with 89% finding the application easy to use. 75% preferred home-based monitoring over inpatient waiting, and 95% rated their overall experience as good, very good or excellent.

## Discussion

This study demonstrates the feasibility, safety and healthcare benefits of implementing a virtual ward for patients awaiting urgent CABG surgery. Across a regional, multisite pathway, early discharge with structured remote monitoring was associated with excellent short-term outcomes, improved system efficiency and high patient satisfaction, without compromising safety. The real-world, multicentre design of this study underscores the scalability and replicability of the model across different hospital settings, increasing its relevance for wider adoption.

### Safety and clinical outcomes

Considered together, zero preoperative MACE while waiting, 0% 30-day mortality (favourable vs national ~0.99%) and a resternotomy rate of 2.34% comparable with national figures point to a clear safety signal for early discharge with structured remote oversight in carefully selected urgent CABG candidates. The pattern suggests perioperative safety was preserved rather than risk being displaced into the preoperative window. In practice, daily symptom prompts with prespecified red flag criteria and same-day clinical review converted alerts into timely reassurance or targeted escalation, maintaining safety while easing inpatient bed pressure.

The digital platform enabled real-time detection of clinical deterioration, generating 65 red alerts from 955 symptom submissions (6.8%). Alerts prompted same-day clinician review, and 9/128 (7.0%) patients had surgery brought forward through established pathways. No patient experienced preventable harm or surgical delay, supporting the platform’s role as a reliable early-warning, triage and escalation mechanism. Importantly, the ability to identify the small subset requiring expedited surgery while safely monitoring the remainder at home gives clinicians confidence to discharge eligible inpatients and deliver remote care while maintaining safety.

Compared with traditional monitoring such as patient-initiated calls or periodic reviews, the virtual ward is proactive: daily structured symptom prompts and prespecified red flag criteria trigger same-day clinician review, generate an auditable record and reduce delays that arise from variable self-reporting or appointment intervals. Non-submission prompts active follow-up, helping to identify access or understanding barriers and to prevent silent deterioration. The standardised workflow supports consistent decision-making and timely escalation, including bringing surgery forward when indicated, while digital time stamps enable service evaluation and transparency.

These findings suggest that selected stable patients, including those with stabilised acute coronary syndrome (ACS), can safely await surgery at home when supported by a proactive virtual ward. However, they do not establish that unmonitored routine discharge would yield similar safety outcomes, as the safety of this model was observed under the conditions of daily structured checks, prespecified red flag review and active follow-up for non-submission.

### System efficiency

The virtual ward model delivered substantial improvements in preoperative system efficiency. Patients spent a median of 10 days at home between hospital discharge and CABG, resulting in an estimated 1152 inpatient bed-days saved across the cohort. Using a coronary care unit bed cost of £917 per night,^9^ this corresponds to a projected cost saving of £1 057 584.

In addition to economic gains, the model enabled the reclassification of many cases from ‘urgent inpatient’ to ‘scheduled elective outpatient’ pathways. This shift alleviates pressure on limited inpatient resources and supports the NHS’s wider elective surgery recovery programme. Managing patients in the community through a monitored virtual ward model avoids the need for prolonged preoperative hospital stays, yet still preserves timely access to surgery. This is evidenced by a median time from angiography to surgery of 13 days, and 10 days from discharge to surgery, demonstrating that transitioning to outpatient care did not delay revascularisation.

While the observed reduction in postoperative length of stay (mean 7.0 days) did not reach statistical significance compared with national data (8.0 days, p=0.084), it signals potential for further downstream benefits, possibly due to better preoperative condition or reduced inpatient deconditioning. Larger studies are needed to clarify this effect.

From a strategic perspective, converting urgent inpatient cases into streamlined elective pathways reduces service bottlenecks, facilitates more efficient theatre scheduling and improves the predictability of resource use. The model supports a more sustainable and patient-centred approach to managing surgical waitlists without compromising clinical safety.

### Scalability and generalisability

This study was delivered across multiple hospital sites using standardised onboarding, remote triage and escalation protocols. This reinforces the model’s feasibility across diverse clinical environments and supports its scalability at national level. The consistency of safety outcomes, platform performance and patient experience across centres suggests that the model is not dependent on a single institution’s infrastructure or personnel. Importantly, the digital platform required minimal daily clinician input (~30 s per patient) and enables asynchronous, batched review, substantially quicker than traditional telephone follow-up, which demands synchronous availability and several minutes per patient, making the approach resource light and sustainable.

### Patient experience

Patient-reported outcomes were overwhelmingly positive. The vast majority (95%) felt safe awaiting surgery at home, 96% reported no surgical delays and 89% found the platform easy to use. These figures support the acceptability of remote pathways, particularly when underpinned by reliable communication and rapid access to care when needed. The preference for home-based monitoring (expressed by 75% of patients) suggests that patient-centred models may improve system flow and the patient journey.

### Limitations

The absence of a randomised control group limits direct causal inference, and comparisons with national averages may be subject to residual confounding. Patients included were clinically stable and capable of engaging with remote technologies, potentially limiting generalisability to more complex or socially vulnerable populations. Nevertheless, this reflects appropriate real-world patient selection for a safety-first deployment of a new pathway.

### Future directions

Building on these promising findings, formal health economic evaluations and randomised controlled trials are needed to quantify the long-term impact on patient outcomes, resource utilisation and cost-effectiveness.

## Conclusion

This multicentre study demonstrates that a virtual ward pathway for patients awaiting urgent CABG is safe, feasible and scalable. It maintained clinical outcomes, reduced preoperative bed occupancy and was highly acceptable to patients. These findings support broader adoption and further evaluation of remote monitoring to enhance cardiac surgical pathways and system efficiency.

## Supplementary material

10.1136/openhrt-2025-003568 online supplemental file 1

## Data Availability

Data are available upon reasonable request.
