# Supplementary material for: Feasibility, safety and outcomes of a virtual ward with remote monitoring for patients awaiting urgent coronary artery bypass graft surgery
Source: Open Heart. 2026 Feb 20;13(1):e003568. doi: 10.1136/openhrt-2025-003568 (PMC12927322; doi:10.1136/openhrt-2025-003568)
Supplement: online supplemental file 1 [file openhrt-13-1-s001.docx]

**Supplementary Material**

**Tables**

**Supplementary Table 1**

| **Criteria** | **Details** |
| --- | --- |
| **Diagnosis** | Inpatients with NSTEMI. unstable angina, or STEMI once the culprit lesion was revascularised |
| **Awaiting CABG** | Patients accepted for surgery |
| **Chest Pain Stability** | Chest pain-free for >48 hours & hospital stay >5 days if troponin positive |
| **Hemodynamic Stability** | No ventricular arrhythmias |
| **Medical Therapy** | On optimal medical therapy |
| **Pre-Surgical Workup** | Agreed and completed before discharge |
| **Remote Access** | Ability to access the Ortus platform |

**Supplementary Table 2**

| **Criteria** | **Details** |
| --- | --- |
| **Coronary Anatomy** | LMS >60% or blocked RCA |
| **Graft Suitability** | Single patent conduit (e.g., LAD with blocked RCA/LCx) |
| **Cardiac Function** | Severe LV impairment |
| **Clinical Stability** | Symptomatic patients (chest pain, SOB, dizziness, palpitations), arrhythmias, decompensated heart failure |
| **Cognitive/Adherence Issues** | Lack of capacity, non-concordance with medication |
| **Home Conditions** | Unsuitable environment or inadequate support |
| **Language Barrier** | No suitable translator at home or telephone services available |

**Supplementary Appendix 1. Cardiac Symptoms At Home QuestionnaireCardiac Symptoms At Home Questionnaire**

**Questions**:

1. **Have you had any chest pain today?**
   - ☐ Yes
   - ☐ No
     If yes, how long did it last? _____________
     What were you doing when it happened? _____________
     Did you feel like passing out? ☐ Yes ☐ No
     Is the pain happening more or less often than yesterday? ☐ More ☐ Less ☐ Same
2. **Have you used your GTN spray today?**
   - ☐ Yes
   - ☐ No
3. **Have you had any palpitations today?**
   - ☐ Yes
   - ☐ No
     If yes, how long did they last? _____________
     Did you feel like passing out? ☐ Yes ☐ No
